# Biochemistry in Endeavor Adventure Racers Study (BEARS)

**DOI:** 10.7759/cureus.1024

**Published:** 2017-02-11

**Authors:** Matthew Wetschler, David Radler, Mark Christensen, Grant Lipman

**Affiliations:** 1 School of Medicine, Stanford University

**Keywords:** wilderness medicine, acute kidney injury, adventure athletes

## Abstract

Background: Adventure sports events consist of a combination of two or more endurance disciplines, such as orienteering, running, and rock climbing, that range from a day’s to a week’s duration. No studies have examined acute kidney injury (AKI) in adventure sports athletes.

Objectives: To describe the prevalence of AKI in participants in the Endeavor Team Challenge, a 30-hour, 40-mile adventure race.

Methods: In this prospective observational study, body weights were recorded at race registration. At the finish line, blood sample results by point-of-care testing and weights were recorded. Changes in serum creatinine (Cr) from an estimated baseline value and severity of AKI were calculated, with “risk of injury” defined as 1.5 x baseline Cr, and “injury” defined as 2 x baseline Cr. These two categories of AKI were combined to calculate the total prevalence.

Results: There were 88 enrolled study participants with complete data available on 46 (52%). The mean age of those enrolled in the study was 36.8 years (+/- 7.7), 90% were males, and body mass index (BMI) was 25.7 kg/m^2^ (+/- 2.4). Of the competitors who completed the study, 34 (73%) had some degree of AKI, with 27 (58%) found to be at "risk" and seven (15%) with "injury". There was a significant correlation between weight loss and elevated Cr (r = -0.29, p = 0.047), with a trend towards nonsteroidal anti-inflammatory drug (NSAID) use being correlated with AKI (p = 0.058).

Conclusion: Acute kidney injury was observed in the majority of the Endeavor Team Challenge adventure racers, similar to what has been observed in multistage ultramarathons, and greater than after standard marathons and single-stage ultramarathons.

## Introduction

Adventure sports are growing in popularity in the United States and around the world with an exponential growth of both participants and events [1]. These events consist of a combination of two or more endurance disciplines, such as orienteering, running, and rock climbing, that may range from a day’s to a week’s duration. The distance usually exceeds the marathon distance of 26.2 miles, placing it in the ultra-endurance category. The Endeavor Team Challenge (ETC) 2014 is a representative event, including over 30 hours of activity and involving more than 40 miles of walking or running interspersed with a variety of other challenges, such as climbing, kayaking, and obstacle courses. 

The presence of acute kidney injury (AKI) in athletes is a well-researched topic in multiple populations: healthy marathon race finishers [2-3], marathon racers seeking medical care [4-5], and ultramarathon race participants [6-7]. Renal dysfunction during endurance events is thought to be due to a multifactorial combination of dehydration, injury from muscle breakdown, heat stress, and the use of non-steroidal anti-inflammatory drugs (NSAIDs), although acute renal failure is rare in this population [8]. No studies have examined AKI in adventure racers as a novel group of athletes. 

The ETC is a unique athletic endeavor; though still predominantly a running event, various other activities are distributed throughout the race, providing continuous activity but not of a single discipline. The interspersed activities between long stretches of running in the ETC may provide an opportunity for normalization of biochemical changes, similar to the pattern observed in running-exclusive multistaged events [6] or following single-day ultramarathons [3, 9]. Alternatively, the multidisciplinary nature of the event may further compound renal dysfunction. The purpose of this study is to determine the prevalence of AKI in adventure racers.

## Materials and methods

### Setting and participants

The Endeavor Team Challenge 2014 took place in Bear Creek, California in the Stanislaus National Forest. It was a 30-hour multidisciplinary event that spanned more than 40 miles and was divided into four subcomponents: a 20-mile march with a weighted pack; a five-event portion involving multiple challenges that included rock climbing, strength tests, rappelling, and obstacle courses; a night navigation portion with waypoint finding (approximately six hours duration), and concluding with a 12-mile run without a pack with a boating component of two miles. All competitors were given an opportunity to enroll in the study during race registration. Institutional approval was obtained from the Stanford University School of Medicine Institutional Review Board (#30928).

### Procedures

The racers were enrolled during race registration the night prior to the race at which time a written informed consent was obtained. Racer demographics, including sex, age, height, weight, and BMI, were recorded. Blood samples were obtained from each participant at the completion of the race. Blood samples were obtained by lancet and blood capillary collection tubes and then immediately measured onsite using an iSTAT point-of-care analyzer (Abbott, East Windsor, NJ).

### Analysis

A descriptive analysis was performed of racer demographics and the prevalence of AKI at the end of the event. Baseline glomerular filtration rate (GFR) was assumed to be 100 mL/min when a racer’s age was less than or equal to 40 years, and 140 minus years of age when participants were older than 40 years [10]. This estimated GFR was then used to calculate a baseline pre-race serum creatinine (Cr) using the “modification of diet in renal disease” (MDRD) equation [11] of GFR = 186 x (Cr)^-1.154^ x (age in years)^-0.203^ x (0.742 if female) x (1.210 if African American). The modified MDRD was used because it is the current formula recommended by the National Kidney Foundation for estimation of renal function [11]. Changes in Cr from an estimated baseline and prevalence of AKI were determined with “risk" of injury defined as 1.5 x baseline Cr, and “injury” defined as 2 x baseline Cr; these two categories of severity were combined to calculate the total prevalence of AKI [12]. The analysis was performed combining all final measured values as a single cohort regardless of whether the participant completed or withdrew from the race. Paired t-test and Wilcoxon signed rank tests were used for analysis; statistical significance was considered to be p < 0.05. All analysis was by the Statistical Package for Social Sciences (SPSS), version 19.0 (IBM SPSS Statistics, Armonk, NY).

## Results

Of the 88 enrolled participants, complete data were available on 46 (52%). Baseline characteristics of all participants are in Table 1. Comparison between those who completed the study and the 48% lost to follow-up showed statistically significant differences in age, BMI, and history of completed marathons. Those who completed the study were, on average, older, had a lower BMI, and had run more marathons. Seventy-four percent of study participants encountered some severity of AKI (Table 2). There was a significant correlation between weight loss and elevated Cr (r = -0.29, p = 0.047), although this was not seen in racers who demonstrated AKI (p = 0.19). There was a trend towards a significant association between NSAID use and racers with AKI (without AKI = 250 mg +/- 173; with AKI = 374 mg +/- 581), although this did not obtain statistical significance (p = 0.058). Differences between those with and without AKI were not statistically significant in regards to percent weight change (0.71 kg, 95% CI -0.36 to 1.78, p = 0.19) or blood urea nitrogen (BUN)/Cr (3.05, 95% CI -0.87 to 6.97, p = 0.12)

**Table 1:** Baseline Characteristics BMI: body mass index; NSAID: non-steroidal anti-inflammatory; Cr: creatinine; SD: standard deviation

Variable	All (n=88)		Completers (n=46)		Lost to Follow Up (n=42)		Comparison of Completers vs Lost to Follow-up
	Mean	SD	Mean	SD	Mean	SD	P-value
Age	36.8	7.7	39	7.5	34.4	7.3	< 0.01
Male (n, %)	79	89.8	42	91.3	37	88.1	0.62
Height (meters)	1.8	0.1	1.8	0.1	1.8	0.1	0.28
Pre-weight	82.6	11.3	81.2	9	84.1	13.3	0.24
BMI	25.7	2.4	25.1	2.3	26.4	2.4	0.02
Marathons (n, %)							< 0.01
0	48	55.2	19	41.3	29	70.7	
1	18	20.7	15	32.6	3	7.3	
2+	21	24.1	12	26.1	9	22	
Ultramarathons (n, %)							0.657
0	69	79.3	35	76.1	34	82.9	
1	9	10.3	6	13	3	7.3	
2+	9	10.3	5	10.9	4	9.8	
NSAID use (n, %)	14	15.9	8	17.4	6	14.3	0.93
Estimated baseline Cr	0.76	0.09	0.77	0.08	0.76	0.09	0.92

**Table 2:** Characteristics by Acute Kidney Injury (AKI) Status and Severity BMI: body mass index; SD: standard deviation

	Normal (n = 12)		At Risk (n = 27)		Injury (n = 7)	
Variable	Mean	SD	Mean	SD	Mean	SD
Age	41.2	8.7	37.5	6.9	41.1	7.3
Male (n %)	12	100%	26	96%	4	57%
Height	1.8	0.1	1.8	0.1	1.8	0.1
BMI	24.4	2.6	25.4	2.2	25.5	2.3
Marathons	1.9	2.4	2.0	3.6	2.6	5.5
Ultramarathons	0.8	1.8	0.4	1.0	0.4	0.8

## Discussion

We noted a greater prevalence of AKI in this adventure race (74%) compared to marathons (40%) [13] and single-stage ultramarathons (34%) [14] but similar to that seen in multistage ultramarathon runners (55-85%) [6-7]. This suggests that continuous multidiscipline events with relatively short running sections can generate similar renal stress to that generated by purely running events. In prior studies of multistage running races, a pattern of near-complete recovery was seen between days of racing [6]. Future studies may define how much rest is required to achieve renal function recovery between intra-race events. All women study participants were categorized in the “injury” or “risk” groups, though the small numbers of female participants (n = 4) limited any statistical inference. Previously, the most commonly reported medical complaints in adventure races were skin and soft tissue injuries [15]. This is in contrast to single-day obstacle races that show more trauma and unique injury patterns [16]. While the occurrence of clinically serious acute renal failure in this population is likely rare, awareness of the high incidence of subclinical AKI is valuable information for race participants.

The mechanism of AKI in our study population is likely multifactorial: a combination of over 30 hours of strenuous, multidiscipline activity rough wilderness terrain and dehydration, heat stress, NSAID use, and muscle tissue breakdown. Because weight loss (a proxy for dehydration) was significantly associated with elevated Cr, we theorize that there is a pathophysiologic contribution of hypovolemia and subsequent decreased renal perfusion as the cause of AKI. This significant association has been previously observed in ultramarathons [7]. Our observed lack of significant association between NSAID use and development of AKI correlates with prior running-specific observational studies [7, 14]. 

As with all convenience sampling, there is a risk that a measured or unmeasured characteristic of study participants may vary from the overall group, introducing unmeasured bias or limiting the study’s generalizability to a broader population. 

We calculated baseline creatinine rather than using direct measurement. This was done to maximize study participants and is common practice in studies similar to this because blood sampling significantly increases study attrition. The attrition in our study was 48%, similar to prior work done in multistage ultramarathons [6]. We used weight gain or loss as a proxy for hydration status. Direct tracking of water intake would have been superior but was too logistically complex to ensure accurate completion. 

Racers dropped out of the study for one of three reasons. They were either unable to complete the competition due to injury or lack of stamina, they refused to have blood sampled, or there was equipment malfunction. We were unable to quantify the reasons in the lost to follow-up group. 

The Endeavor Team Challenge is a unique event, and although it is an example of an adventure race, there is significant heterogeneity within the adventure race domain. Observed trends during the ETC may be more or less apparent given the length, types of activity, and climate of other events.

## Conclusions

Acute kidney injury was observed in the majority of Endeavor Team Challenge adventure racers, similar to that seen with multistage ultramarathons and greater than that seen with standard marathons and single-stage ultramarathons. Given that multistage endurance events are becoming more popular, studies to delineate how much time is required for adequate renal function recovery are needed and could potentially help race planning for optimal safety. Because of the high prevalence of NSAID use during endurance events, further studies are recommended to examine the impact of NSAIDs on renal function in the setting of multistage events.
