# Correction: Biochemistry in Endeavor Adventure Racers Study (BEARS)

**DOI:** 10.7759/cureus.c9

**Published:** 2017-06-01

**Authors:** Matthew Wetschler, David Radler, Mark Christensen, Sean Rundell, Grant Lipman

**Affiliations:** 1 School of Medicine, Stanford University; 2 Department of Rehabilitation Medicine, University of Washington

**Title:** Biochemistry in Endeavor Adventure Racers Study (BEARS)

**Original, Incorrect Author List:** Matthew Wetschler, David Radler, Mark Christensen, Grant Lipman 

**Original Citation:** Wetschler M, Radler D, Christensen M, et al. (February 11, 2017) Biochemistry in Endeavor Adventure Racers Study (BEARS). Cureus 9(2): e1024. doi:10.7759/cureus.1024

The submitting author erroneously omitted the fourth author, Sean Rundell. This mistake was only just noticed by the authors. The author list should be corrected as follows. The original, incorrect author list will be changed.

**Corrected Author List: **Matthew Wetschler, David Radler, Mark Christensen, Sean Rundell, Grant Lipman 

